# Prediction of Specific Anxiety Symptoms and Virtual Reality Sickness Using In Situ Autonomic Physiological Signals During Virtual Reality Treatment in Patients With Social Anxiety Disorder: Mixed Methods Study

**DOI:** 10.2196/38284

**Published:** 2022-09-16

**Authors:** Joo Young Chun, Hyun-Jin Kim, Ji-Won Hur, Dooyoung Jung, Heon-Jeong Lee, Seung Pil Pack, Sungkil Lee, Gerard Kim, Chung-Yean Cho, Seung-Moo Lee, Hyeri Lee, Seungmoon Choi, Taesu Cheong, Chul-Hyun Cho

**Affiliations:** 1 School of Industrial and Management Engineering Korea University Seoul Republic of Korea; 2 Department of Psychiatry Chungnam National University Sejong Hospital Sejong Republic of Korea; 3 School of Psychology Korea University Seoul Republic of Korea; 4 Department of Biomedical Engineering Ulsan National Institute of Science and Technology Ulsan Republic of Korea; 5 Department of Psychiatry Korea University College of Medicine Seoul Republic of Korea; 6 Department of Biotechnology and Bioinformatics Korea University Sejong Republic of Korea; 7 Department of Software Sungkyunkwan University Suwon Republic of Korea; 8 Department of Computer Science and Engineering Digital Experience Laboratory Korea University Seoul Republic of Korea; 9 Department of Film & Multimedia Korea National University of Arts Seoul Republic of Korea; 10 Department of Computer Science and Engineering Pohang University of Science and Technology Pohang Republic of Korea

**Keywords:** social anxiety, virtual reality, autonomic physiological signals, machine learning, virtual reality sickness

## Abstract

**Background:**

Social anxiety disorder (SAD) is the fear of social situations where a person anticipates being evaluated negatively. Changes in autonomic response patterns are related to the expression of anxiety symptoms. Virtual reality (VR) sickness can inhibit VR experiences.

**Objective:**

This study aimed to predict the severity of specific anxiety symptoms and VR sickness in patients with SAD, using machine learning based on in situ autonomic physiological signals (heart rate and galvanic skin response) during VR treatment sessions.

**Methods:**

This study included 32 participants with SAD taking part in 6 VR sessions. During each VR session, the heart rate and galvanic skin response of all participants were measured in real time. We assessed specific anxiety symptoms using the Internalized Shame Scale (ISS) and the Post-Event Rumination Scale (PERS), and VR sickness using the Simulator Sickness Questionnaire (SSQ) during 4 VR sessions (#1, #2, #4, and #6). Logistic regression, random forest, and naïve Bayes classification classified and predicted the severity groups in the ISS, PERS, and SSQ subdomains based on in situ autonomic physiological signal data.

**Results:**

The severity of SAD was predicted with 3 machine learning models. According to the F1 score, the highest prediction performance among each domain for severity was determined. The F1 score of the ISS mistake anxiety subdomain was 0.8421 using the logistic regression model, that of the PERS positive subdomain was 0.7619 using the naïve Bayes classifier, and that of total VR sickness was 0.7059 using the random forest model.

**Conclusions:**

This study could predict specific anxiety symptoms and VR sickness during VR intervention by autonomic physiological signals alone in real time. Machine learning models can predict the severe and nonsevere psychological states of individuals based on in situ physiological signal data during VR interventions for real-time interactive services. These models can support the diagnosis of specific anxiety symptoms and VR sickness with minimal participant bias.

**Trial Registration:**

Clinical Research Information Service KCT0003854; https://cris.nih.go.kr/cris/search/detailSearch.do/13508

## Introduction

Anxiety involves uncertainty about the expectancy of a threat. It is a normal emotional response that helps individuals heed and cope with potential signs of danger [1]. Anxiety induces anticipatory stress and various neurophysiological responses. Previous research has shown that certain brain regions that process threatening information can biologically trigger stress response systems, such as the autonomic nervous system and hypothalamic-pituitary-adrenal axis. Hyperactivity of the autonomic response can induce the expression of anxiety signals, perception of sensations from inside the body, and central periphery interactions [2].

Social anxiety disorder (SAD) is the fear of negative evaluation and embarrassment in social situations. For instance, anxiety can occur when thinking about being observed while eating and speaking publicly [3]. The physiological symptoms of SAD include heart palpitations, sweating, tremors, shaking, and blushing. Many existing studies have focused on anxiety-induced heart reactions, and have discovered that anxiety increases blood catecholamine concentrations [4] and causes excess sympathetic activation and parasympathetic withdrawal [5].

Existing treatment methods for SAD include pharmacotherapy and psychotherapy, such as cognitive behavioral therapy (CBT) [6]. During CBT, an exposure technique allows the patient to experience and participate in a feared situation to create a natural process related to fear reduction [7]. However, individuals with social anxiety rarely request for support from professionals owing to fear of the stigma of mental health treatment. Therefore, the demand for accessibility to CBT via online content has been increasing. Moreover, the range of virtual reality (VR) treatments is expanding [8].

Recent VR intervention research has created virtual environments, such as restaurants. Individuals undergoing VR intervention showed reduced anxiety regarding daily social interactions. They also showed a decrease in social interaction anxiety and depression, and improved satisfaction with daily life after the VR intervention [9].

Exposure therapy is the mainstay VR treatment for SAD. Measuring anxiety symptoms in real time while applying the exposure technique enables the confirmation of actual SAD symptom levels and the appropriate application of personalized therapeutic methods.

However, VR sickness influences user experience when using VR systems [10]. The symptoms of VR sickness include eye fatigue, disorientation, and nausea [11]. Sickness resulting from VR is referred to by many names, including motion sickness, cybersickness, and simulator sickness. These uncomfortable feelings disturb VR intervention; therefore, VR sickness is a crucial problem that must be solved. The most popular method to assess VR sickness is the Simulator Sickness Questionnaire (SSQ) [12]. The symptoms of simulator motion sickness resemble those of motion sickness, but are less severe. Three distinct symptom groups, namely nausea, oculomotor, and disorientation, were clustered across 16 symptom variables. Because simulator sickness–related symptoms differ from motion sickness patterns, a measurement system was established through this grouping. The SSQ scores based on factor analysis models provide a scale score as a good indicator of overall simulator sickness severity. This quantifies simulator sickness for activities that can lead to symptoms. However, the method cannot be used for real-time measurements because it measures psychological scales through a questionnaire after VR intervention. There are several technical approaches for overcoming VR sickness, and measuring and evaluating it in real time is crucial. This is one of the factors determining the difference in individual sensitivity and future compliance. If it is not addressed during VR intervention, patient compliance can be greatly reduced [13].

Several studies have proposed interventions for social anxiety using VR treatment technology. The results showed that social interaction anxiety and quality of life improved when treatment was applied [9]. In particular, previous studies have shown that exposure to scenarios of various social situations through VR is effective for alleviating social anxiety symptoms [14].

Recently, a study applied VR treatment to anxiety disorders, such as SAD [15], proposed a VR intervention for SAD treatment, and verified its clinical effect by reducing scores on the Social Anxiety Disorder Scale. This method included CBT to enable participants to overcome SAD by exposing them to a self-introduction situation. The treatment had exposure therapy for patients with SAD through VR for over 6 sessions. Compared with previous studies that applied relatively unidirectional VR content, this study applied a customized approach that divided the difficulty according to the patient’s own symptom report. However, this study used a subjective self-assessment scale, which was influenced by patient bias. Furthermore, survey-oriented progress is lengthy and may have different results based on subjective judgment.

We have attempted to investigate SAD in relation to VR treatment using objective data such as brain imaging data [16]. Objective physiological signals, which reflect the degree of anxiety symptoms or sickness symptoms, can be assessed in real time during VR treatment sessions by several sensors. This would provide a basis for stand-alone interactive content to be provided during VR treatment for anxiety. Moreover, reducing VR motion sickness could help enhance user experience and compliance using physiological signals.

In this study, we developed a machine-learning model to predict the severity of specific anxiety symptoms and VR sickness using physiological signal data that were measured in situ during VR treatment sessions in patients with SAD.

## Methods

### Participants

Participants were recruited through advertisements on the universities’ online sites. The inclusion criteria for the SAD group were set as follows: (1) men and women aged between 19 and 31 years using the Korean language; (2) condition meeting the Diagnostic and Statistical Manual of Mental Disorders, fourth edition (DSM-IV) criteria for SAD, as evaluated by the Mini-International Neuropsychiatric Interview [17]; (3) no psychiatric comorbidity, except for depression and panic disorder, and no experience in psychotropic drug treatment; (4) not currently receiving psychotherapy; (5) not currently diagnosed with medical or neurological disorders; (6) no history of psychotic symptoms that can be triggered by VR interventions; and (7) no susceptibility to visual stimuli. We set the exclusion criteria as follows: (1) any history of organic brain damage or intellectual disability; (2) a history of psychotic symptoms that can be triggered by VR interventions; (3) vulnerability to visual stimuli; and (4) ineligibility for participation in a magnetic resonance imaging assessment (during the study, we performed a functional magnetic resonance imaging evaluation for another project in the same subjects) [18].

Forty individuals with SAD participated in the study. Among them, 8 individuals quit for personal reasons (eg, time limitations). Ultimately, 32 participants completed the study. All participants completed all VR sessions. The number of people who participated in the entire session was 32, but 6 of them, for whom the sensor data for a specific session were not collected because of problems such as sensor errors, were excluded from data analysis. Consequently, data from 26 individuals were used for specific anxiety symptom and VR sickness predictions after the data qualification process.

### Ethics Approval

The study was approved by the Institutional Review Board of Korea University Hospital (2018AN0377). It was conducted in accordance with the principles of the Declaration of Helsinki. All participants were informed of the study procedure and provided written informed consent before the experiment. This study was registered on the Clinical Research Information Service (KCT0003854).

### Study Design

#### VR Intervention for SAD

**Figure 1:**
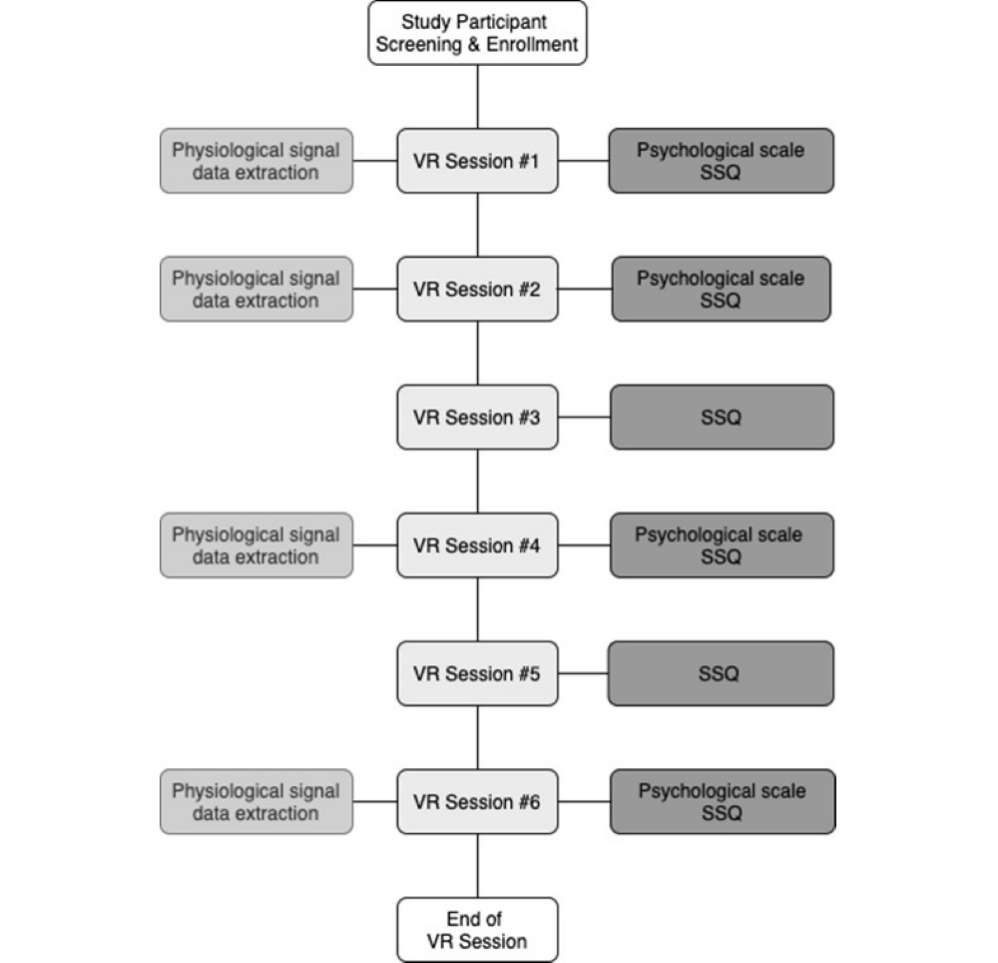
Virtual reality (VR) treatment process with psychological scale measurement and physiological signal data extraction. Psychological scale assessments included Beck Anxiety Inventory, State-Trait Anxiety Inventory, Social Phobia Scale, Social Interaction Anxiety Scale, Brief-Fear of Negative Evaluation Scale, Internalized Shame Scale, Post-Event Rumination Scale, and Liebowitz Social Anxiety Scale. SSQ: Simulator Sickness Questionnaire.

The study design included VR sessions, psychological scale evaluations, and in situ autonomic physiological signal data measurements (Figure 1). A total of 6 VR sessions and 4 psychological scale assessments were performed. A recent study found that the amount of anxiety that increased during actual conversation and the anxiety that increased during a conversation in a VR environment was similar [19]. In a study comparing VR exposure therapy and real-world exposure therapy for SAD [20], 97 participants with a major diagnosis of SAD were randomly assigned to VR exposure therapy, real-world exposure therapy, or a waiting list. A standardized self-report scale showed a statistically significant improvement in those who completed active treatment than in those on the waiting list. Thus, VR exposure therapy was as effective as real-world exposure therapy for treating SAD. In this study, participants completed VR treatment sessions that included interactive content on social anxiety situations, and the VR intervention was performed face-to-face and provided individually. The intervention proceeded in a VR environment, and each participant entered a meeting room with several nonplaying characters [15]. The content had introduction, core, and finishing stages. In the introduction phase, participants selected their avatars and were informed how to manipulate the VR device to progress through the stages [21]. Participants performed a warm-up session with mediation to support their adaptation. The core stage had an intervention in which college student’s participated in social situations where they introduced themselves to each other. Participants proceeded with the treatment session by selecting the difficulty of the core level as easy, medium, or hard. In the first session, all participants started the VR intervention at the easy level. From the second session, participants freely chose the level of difficulty they wanted based on the difficulty they experienced during the treatment process. This is related to the degree of difficulty in interacting in social situations. The reaction of the nonplaying characters was based on the difficulty level selected in the previous stage. In the easy-level session, the nonplaying characters concentrated well on the patients’ self-introduction. Nonplaying characters appeared distracted in the hard level, such as yawning or engaging in chatting. The finishing stage provided cognitive and behavioral safety guidelines for SAD with text and audio information during the VR intervention. The VR headset used was VIVE (HTC Corporation). The in situ autonomic physiological signals (heart rate [HR] and galvanic skin response [GSR]) of the participants were measured during the VR experience.

#### Data Collection and Separation

This study analyzed specific anxiety symptoms and in situ autonomic physiological signal data. For this purpose, physiological signal data were measured in real time during 4 VR sessions (#1, #2, #4, and #6), where psychological scale tests were performed after VR treatment. Data were extracted as independent variables. The SSQ was evaluated during all treatment sessions; therefore, in situ physiological signal data for all sessions were extracted. We created a data frame with the HR and GSR data. Values ​​below zero in the HR and GSR measurements were considered outliers. Outliers were removed, and a moving average was used to reduce the noise of the data.

### Measurement

#### Psychological Scales

We measured psychological scales using subjective surveys after the VR sessions #1, #2, #4, and #6. The Beck Anxiety Inventory [22], State-Trait Anxiety Inventory [23], Social Phobia Scale [24], Social Interaction Anxiety Scale [25], Brief-Fear of Negative Evaluation Scale [26], Internalized Shame Scale (ISS) [27], and Post-Event Rumination Scale (PERS) [28] were used. After building a simple linear regression model for the scales, their subdomains, and the SSQ, the best performing group of scales based on the mean squared error of the models’ prediction results was analyzed. Subsequently, we generated a machine learning model to predict the survey results. The linear regression coefficient was included as an independent variable for prediction; therefore, the specific anxiety symptoms were selected in the order of the smallest root mean square error deviation when predicting with simple linear regression. The subdomains of the corresponding specific anxiety symptoms were also included and used for prediction. The ISS and PERS showed a good reflection of the physical response to SAD. In this study, the machine learning model used the ISS and PERS as independent variables. Multimedia Appendix 1 provides the complete regression results for our machine learning model.

The ISS assesses the patients’ shame and self-esteem [27]. It has 30 items that measure shame and self-esteem (24 and 6 items, respectively). ISS data were measured using the ISS written in the Korean language [29]. Factor analysis involved the following 4 factors: inadequacy, emptiness, self-punishment, and fear of mistakes, with 10, 5, 5, and 4 items, respectively.

The PERS measures postevent ruminations during social situations [28,30]. It comprises 2 scales, negative and positive rumination, with 5 and 9 items, respectively. Each answer was measured on a numerical scale from 0 (low score) to 4 (high score), with a higher score indicating that the individual frequently experiences rumination.

#### VR Sickness Scale

The SSQ was developed for VR sickness, and is the gold standard for assessing physical sickness after exposure to a simulator or VR environment [12]. The SSQ can measure 16 symptoms of VR sickness. The subdomains are grouped into nausea (general discomfort, increased salivation, sweating, nausea, difficulty concentrating, stomach awareness, and burping), oculomotor (general discomfort, fatigue, headache, eyestrain, difficulty focusing, difficulty concentrating, and blurred vision), and disorientation (difficulty focusing, nausea, fullness of the head, blurred vision, dizziness with eyes open, dizziness with eyes closed, and vertigo). The total VR sickness score was the sum of nausea, oculomotor, and disorientation group scores, with a higher score indicating a greater level of simulator sickness.

### Data Labeling and Processing

We used k=2 to categorize the specific anxiety symptoms in the severe and nonsevere groups, where k is the number of median points ​​of the clusters to be classified. Data are grouped as per the number of hyperparameters k. In k-means clustering, the algorithm can be executed when the user determines the number of clusters. K-means clustering is a representative detached clustering algorithm that uses unsupervised learning [31].

We labeled the severe and nonsevere groups using k-means clustering for each specific anxiety symptom and VR sickness. The ISS cutoff values for dividing the severe groups were 48 (total score), 11 (mistake anxiety), 9 (self-punishment), 10 (emptiness), and 16 (inappropriate). The PERS cutoff values were 41 (total score), 17 (positive), and 27 (negative). For the SSQ, severity classification was performed using k-means clustering via the sum of the response scores for each category. The cutoff values for dividing the severe group were 9 (total score), 7 (nausea), 8 (oculomotor), and 4 (disorientation).

### Physiological Signal Data

We measured all participants’ HR and GSR during the VR sessions in real time. These values are closely related to in situ physiological signaling responses related to anxiety. We recorded the data using a Shimmer3 GSR+ Unit (Shimmer) with 3 channels. Two channels tracked the electrodermal skin conductance level signals via hand electrodes attached to the first and middle fingers. We monitored the fingers of the nondominant hand at a sampling frequency of 52 Hz. The third channel recorded cardiac volume data from an infrared sensor attached to the left earlobe. Data were converted into HR data using a software system.

Preprocessing removed any sections where the HR and GSR were not measured or had negative values. The noise was reduced using the moving average method at 2-s intervals. We extracted all the data from the time series data set of the participants. We also calculated the mean, standard deviation, minimum, maximum, linear regression coefficient, maximum difference, peak ratio, and mean difference for each in situ physiological signal. These created the data frame. To calculate the peak ratio [32], we divided the number of peaks by the length of the physiological signal data.

We used real data for the prediction model; therefore, generating data was a challenge. The severe and nonsevere groups varied in size; therefore, we manipulated the data set to improve the model performance. We did not consider the undersampling technique because the number of participants was insufficient. Conversely, we used the oversampling technique on the minority group to improve classifier performance.

The SMOTE (Synthetic Minority Oversampling Technique) approach generated insufficient data [33]. This method helps construct classifiers from imbalanced data sets (where the classification categories are not approximately equally distributed), such as real-world data sets with a small number of abnormal cases.

### Statistical Analysis

#### Group-Based Analysis

Differences between groups (severe and nonsevere) were assessed using independent *t* tests in Python with SciPy version 1.5.2. Differences were considered statistically significant at *P*<.05. Furthermore, we analyzed correlations between the extracted variables. The variables with the strongest positive correlations were mean and peak GSR ratios. The variables with the second strongest positive correlations were the mean and standard deviation of the GSR. The variables with the strongest negative correlations were the linear regression coefficient and standard deviation of the GSR. The second most negatively correlated variables were the GSR difference and HR linear regression coefficient. All correlations are illustrated in Multimedia Appendix 2. There was a high correlation between the mean and peak GSR (0.92); however, removing this feature decreased the prediction score. Therefore, this feature was retained. All other correlations were <0.86.

#### Machine Learning Techniques

All machine learning techniques were performed using Python version 3.7.7. Logistic regression [34], random forest [35], and naïve Bayes [36] classification were performed using Scikit-learn version 0.23.2. The random forest model avoids overfitting, thereby improving model accuracy. Additionally, we can select relatively important ranks of variables in the classification model. The naïve Bayes model is a conditional probability-based classification method that calculates the probability of features belonging to each class and handles noise and missing values reasonably.

In addition, when training, it works excellently regardless of the size of the data, and it is easy to obtain the estimated probabilities for prediction. Although 32 samples of data could be insufficient, we amplified the patients’ data using SMOTE methodology after segmentation for each session. We used machine learning models that can perform reasonable prediction with a few data points to minimize performance degradation because of insufficient data. We calculated the accuracy, F1 score (weighted average of precision and recall), and area under the curve (AUC) for the prediction.

All machine learning models, except for the random forest model, were performed using the oversampling technique on the training data set. This supplemented insufficient data after the training-test split. We performed 5-fold cross-validation for the random forest model to improve the prediction score. During the prediction, sampling of the severe and nonsevere groups was stratified for training-test splits to ensure equally frequent severity.

## Results

### Classified Specific Anxiety Symptoms and VR Sickness

Specific anxiety symptom and VR sickness results were compared between the severe and nonsevere groups using k-means clustering. The smallest difference between group sizes was observed for ISS self-punishment (severe vs nonsevere group, 53 vs 51). Based on the average score of the specific anxiety symptoms, the most significant difference between the groups was the ISS score (severe vs nonsevere group, 59.951 vs 33.222). ISS emptiness showed the smallest difference between groups (severe vs nonsevere group, 12.91 vs 5.75). After labeling with k-means clustering, the anxiety symptom with the lowest distortion was ISS mistake anxiety and that with the highest distortion was total ISS (1.263 vs 7.439).

After 4 VR sessions with 26 participants, 104 sets of VR sickness data were collected. These were labeled as severe and nonsevere groups using k-means clustering. Distortion of k-means clustering was 2.57 for total VR sickness, 1.44 for nausea, 1.71 for oculomotor, and 1.16 for disorientation. Table 1 presents the statistical analysis results of the severe and nonsevere groups. The boxplot for the severe and nonsevere groups is illustrated in Multimedia Appendix 3.

**Table 1:** Severe and nonsevere groups clustered by k-means clustering.

Measure	Groups^a^					Minimum value		Cutoff value		Maximum value		Distortion^b^
	Nonsevere group count	Severe group count	Nonsevere group mean	Severe group mean								
ISS^c^	63	41	33.222	59.951	3		<48		92		7.439	
ISS mistake anxiety	34	70	8.176	12.957	1		<11		16		1.263	
ISS self-punishment	51	53	4.961	12.453	1		<9		20		1.835	
ISS emptiness	60	44	5.750	12.910	0		<10		20		2.104	
ISS inappropriate	58	46	9.155	21.978	2		<16		36		3.344	
PERS^d^	47	57	32.766	48.140	19		<41		64		4.203	
PERS positive	36	68	10.059	21.000	2		<17		35		3.007	
PERS negative	50	54	16.900	36.016	3		<27		56		5.163	
Total VR^e^ sickness	73	31	3.247	13.710	0		<9		29		2.568	
Nausea group	98	6	1.786	10.500	0		<7		15		1.445	
Oculomotor group	63	41	2.238	8.341	0		<6		15		1.709	
Disorientation group	69	35	0.986	6.000	0		<4		12		1.164	

^a^After labeling into severe and nonsevere groups through k-means clustering, the numerical characteristics and differences between the groups are shown for each group.

^b^Distortion was calculated using the k-means clustering model with k=2.

^c^ISS: Internalized Shame Scale.

^d^PERS: Post-Event Rumination Scale.

^e^VR: virtual reality.

#### Differences in Physiological Signals According to Specific Anxiety Symptom Severity

There were significant differences in the GSR min between the severe and nonsevere groups stratified by the ISS (*t*_102_=1.39; *P*=.17). In addition, groups stratified by the ISS mistake anxiety showed a significant difference in the HR peak ratio (*t*_102_=2.07; *P*=.04) and GSR average change (*t*_102_=2.02; *P*=.046). Groups stratified by ISS self-punishment showed significantly different GSR min values (*t*_102_=2.34; *P*=.02). Groups stratified by ISS emptiness showed a difference in mean HR (*t*_102_=−2.44; *P*=.02). Groups stratified by ISS inappropriate showed a significant difference in mean HR (*t*_102_=−2.23; *P*=.03). Groups stratified by PERS had significantly different mean HRs (*t*_102_=−1.99; *P*=.050). Furthermore, groups stratified by PERS positivity showed significantly different mean HRs (*t*_102_=2.51; *P*=.01), HR min values (*t*_102_=2.49; *P*=.01), and HR peak ratios (*t*_102_=2.15; *P*=.03). Groups stratified by PERS negative had significantly different mean HRs (*t*_102_=−2.79; *P*=.006), HR min values (*t*_102_=−2.49; *P*=.01), HR peak ratios (*t*_102_=−2.15; *P*=.03), and GSR max values (*t*_102_=−2.00; *P*=.048). The severe and nonsevere groups by anxiety symptoms are illustrated in Multimedia Appendix 4.

We performed *t* tests after labeling the severity of VR sickness, for each VR sickness subdomain. The most significant difference in total VR sickness was for the HR min value (*t*_102_=−1.63; *P*=.02). In the nausea group, the HR max value was significantly different between the groups (*t*_102_=−2.47; *P*=.02). Additionally, the oculomotor group differed in the HR min value (*t*_102_=−2.19; *P*=.03). The disorientation group showed a difference in total HR change (*t*_102_=−2.14; *P*=.04) and total GSR change (*t*_102_=−2.09; *P*=.04). Differences in the severe and nonsevere groups according to VR sickness are illustrated in Multimedia Appendix 5.

### Specific Anxiety Symptom and VR Sickness Prediction Based on the Physiological Signal Data

After oversampling the minority data using SMOTE, the F1 score [37], accuracy, and area under the receiver operating characteristic curve were calculated after predicting the survey results using logistic regression analysis, random forest, and naïve Bayesian methodology. Additionally, we calculated the feature importance of specific anxiety symptoms. The variable importance can be calculated based on the impurity index [38]. We used the feature importance of a random forest–based prediction model.

#### Anxiety Symptom Prediction

The classifications of specific anxiety symptom results were analyzed based on 3 models. Logistic regression [34], random forest [35], and naïve Bayes classifier [36] analyzed the severity of specific anxiety symptoms for participants. The data were amplified using the SMOTE method to prevent performance degradation due to unbalanced data.

The classification performance was measured using the F1 score. The F1 scores of the ISS mistake anxiety subdomain were 0.8421, 0.7368, and 0.7647, as calculated by logistic regression, random forest, and naïve Bayesian classifier, respectively. These values were higher than those of the other ISS subdomains. The classification performance of the PERS negative subdomain was relatively higher than that of the PERS positive subdomain, with 0.6667, 0.6154, and 0.6452 as measured by logistic regression, random forest, and naïve Bayes classifier, respectively.

Specifically, we classified the ISS using a logistic regression model with an F1 score of 0.7619. The logistic regression model revealed an F1 score of 0.8421 for ISS mistake anxiety, whereas the naïve Bayes classification model revealed a score of 0.7857 for ISS self-punishment. The logistic regression model revealed an F1 score of 0.6429 for ISS emptiness and 0.7200 for ISS inappropriate. The random forest model revealed an F1 score of 0.7568 for PERS. Further, the naïve Bayes classification model showed an F1 score of 0.7619 for PERS positivity. Finally, the random forest model revealed an F1 score of 0.7097 for PERS negativity. The specific anxiety symptom prediction results are illustrated in Table 2 and Multimedia Appendix 6.

**Figure 2:**
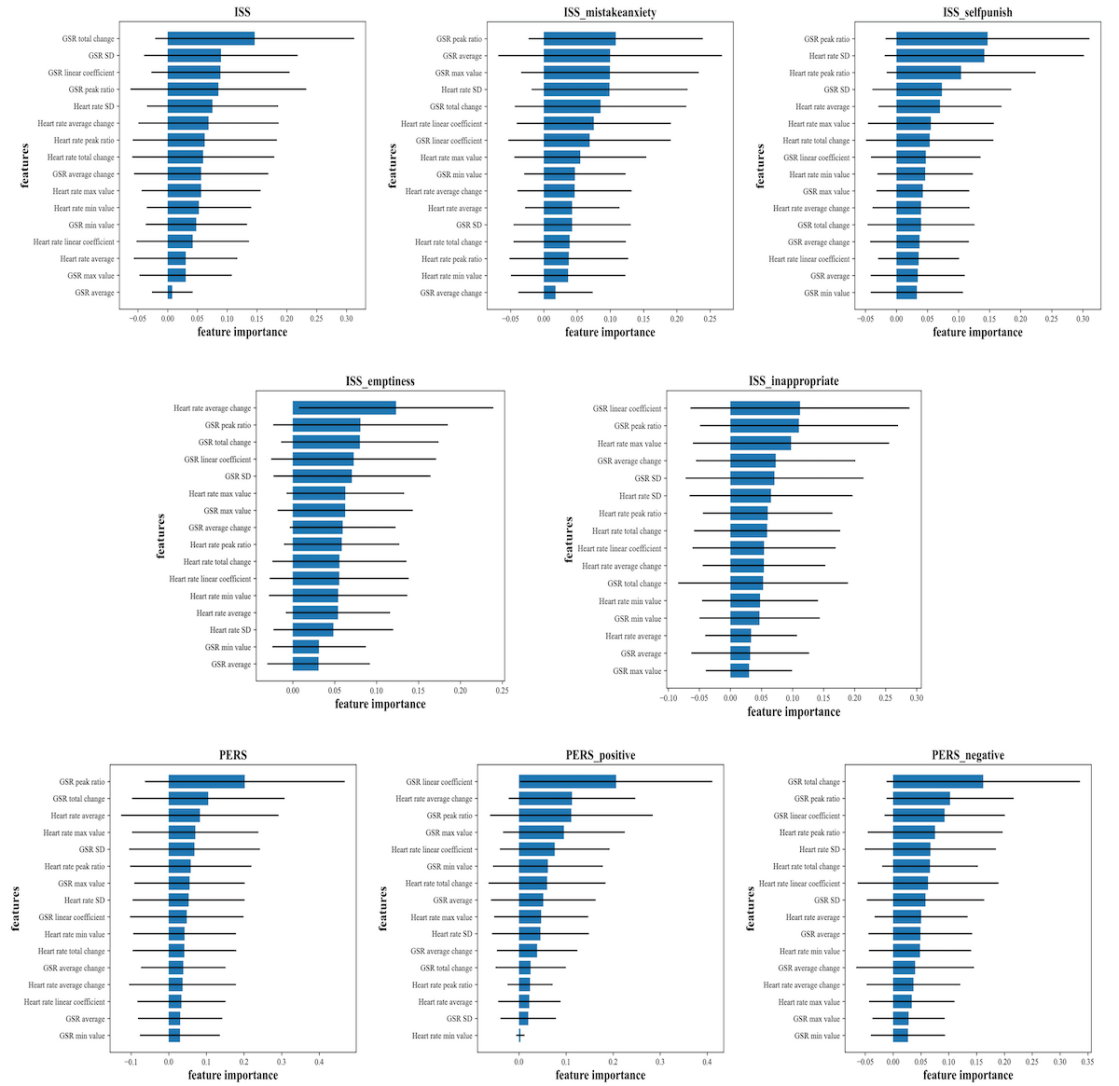
Feature importance of specific anxiety symptoms (random forest model). After predicting the subdomains of each anxiety symptom using the random forest model, the feature importance for each model was calculated and sorted in descending order. GSR: galvanic skin response; ISS: Internalized Shame Scale; PERS: Post-Event Rumination Scale.

For ISS, ISS mistake anxiety, ISS self-punishment, ISS inappropriate, PERS, PERS positive, and PERS negative, physiological signal data related to GSR had the highest importance, and for ISS emptiness, physiological signal data related to HR had the highest importance. The feature importance of specific anxiety symptoms in the random forest model is illustrated in Figure 2.

**Table 2:** Specific anxiety symptom classification model evaluation with F1 score, accuracy, and area under the curve.

Variable^a^	Logistic regression			Random forest				Naïve Bayes classifier		
	F1 score	Accuracy	AUC^b^	F1 score	Accuracy	AUC	F1 score		Accuracy	AUC
ISS^c^	0.762	0.808	0.827	0.632	0.731	0.706	0.727		0.769	0.775
ISS mistake anxiety	0.842	0.769	0.733	0.790	0.692	0.608	0.765		0.692	0.660
ISS self-punishment	0.750	0.692	0.673	0.727	0.769	0.769	0.786		0.769	0.769
ISS emptiness	0.643	0.615	0.625	0.571	0.654	0.639	0.600		0.692	0.673
ISS inappropriate	0.720	0.731	0.732	0.571	0.654	0.639	0.714		0.692	0.702
PERS^d^	0.667	0.654	0.676	0.757	0.654	0.625	0.667		0.615	0.607
PERS positive	0.615	0.615	0.630	0.500	0.769	0.660	0.762		0.808	0.861
PERS negative	0.667	0.654	0.654	0.710	0.654	0.654	0.645		0.577	0.577

^a^After predicting the severity of each specific anxiety symptom using logistic regression, random forest, and naïve Bayes classifier models, the performance of each model was evaluated.

^b^AUC: area under the curve.

^c^ISS: Internalized Shame Scale.

^d^PERS: Post-Event Rumination Scale.

#### VR Sickness Prediction

We classified the severity of total VR sickness with an F1 score of 0.7059, that in the nausea group with a score of 0.4000, that in the oculomotor group with a score of 0.6667, and that in the disorientation group with a score of 0.6364. The classification performance for the nausea group subdomain had the highest AUC of 0.94. The VR sickness results are illustrated in Table 3 and Multimedia Appendix 7.

**Figure 3:**
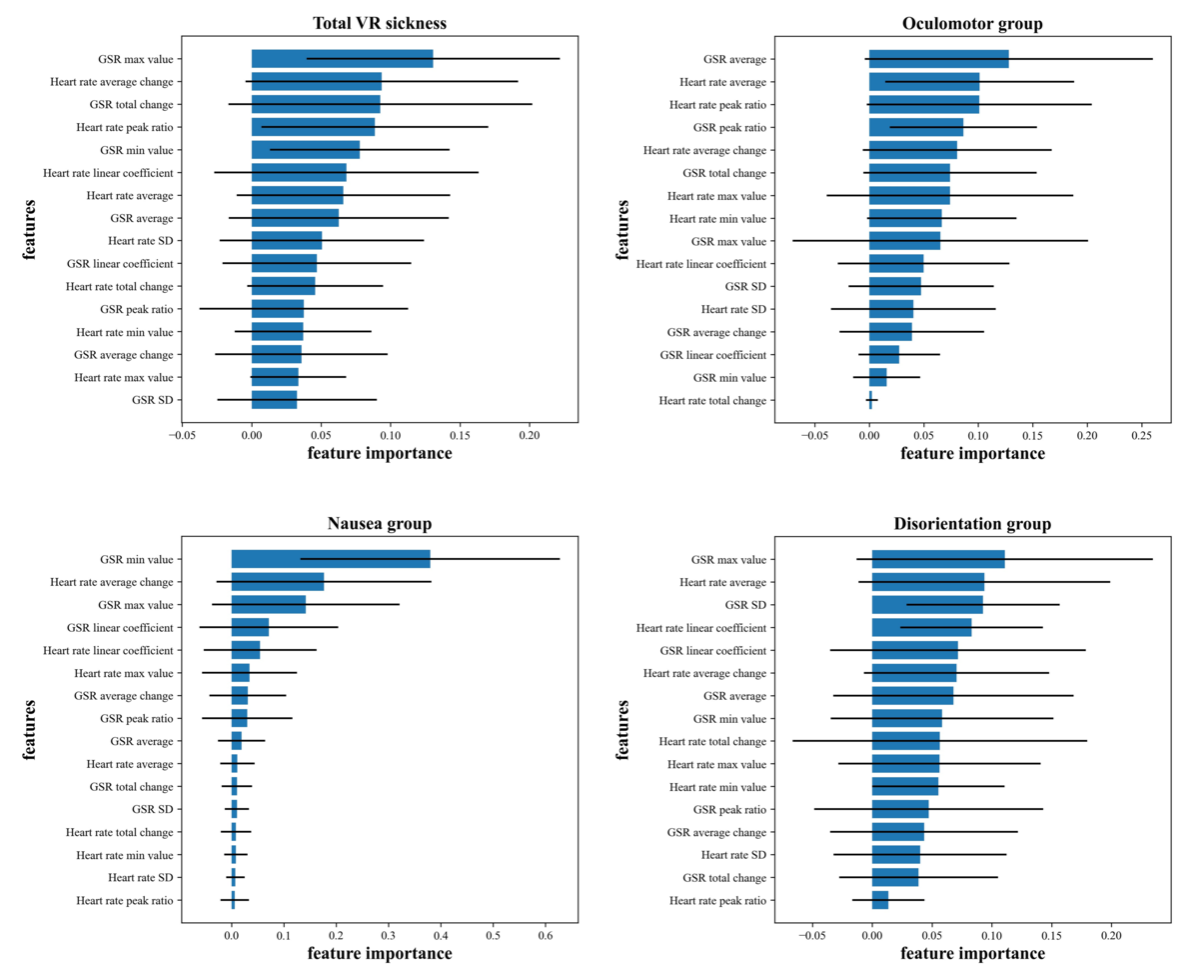
Feature importance of virtual reality (VR) sickness (random forest model). After predicting the subdomains of each VR sickness scale using the random forest model, the feature importance for each model was calculated and sorted in descending order. GSR: galvanic skin response.

When feature importance was calculated for the classification of the severity of VR sickness for each type, physiological signal data related to GSR were selected as essential variables for all subdomains. All subdomains of VR sickness symptoms showed HR-related features as the second most crucial factor in variation. Figure 3 illustrates the importance of VR sickness features from the random forest model.

**Table 3:** Virtual reality sickness classification model evaluation with F1 score, accuracy, and area under the curve.

Variable^a^	Random forest		
	F1 score	Accuracy	AUC^b^
Total VR^c^ sickness	0.7059	0.8077	0.7917
Nausea group	0.4000	0.8846	0.9400
Oculomotor group	0.6667	0.6538	0.7000
Disorientation group	0.6364	0.6923	0.7124

^a^After predicting the severity of each VR sickness scale using the random forest model, the model’s performance was evaluated.

^b^AUC: area under the curve.

^c^VR: virtual reality.

## Discussion

### Principal Findings

We developed and tested machine learning models to predict specific anxiety symptoms and VR sickness for SAD using in situ autonomic physiological signal data measured during participatory and interactive VR treatment. The severity of specific anxiety symptoms and the side effects of VR treatment are an essential part of digital therapy. Usually, these kinds of evaluations rely on subjective reporting by patients. However, autonomic physiological responses, such as blushing, sweating, and shivering, could play a central role in assessing symptoms [39]; therefore, they have the potential to be used for monitoring various symptoms related to anxiety or VR sickness.

Previous studies have assessed the relationship between physiological cues and SAD. However, real-time analysis is limited because of current sensor technology and evaluation methods [40]. Furthermore, individuals with SAD cannot be treated because of methodological problems [41-43].

VR treatment techniques extend beyond the traditional psychiatric therapeutic approach [44]. To create a therapeutic VR system, we must build an interactive system rather than simply provide a VR environment. Interactive VR may have several benefits for psychiatric disorders in terms of treatment. First, it enhances ecological validity by immersing individuals in emotionally engaging virtually constructed therapeutic environments. Second, it can be flexibly used to present patients in various contexts, enabling personalized treatment according to the assessment of individual differences in symptoms during VR treatment [45]. We expect our findings to be useful in interactive VR treatment, especially for specific anxiety symptoms and VR sickness.

We showed that the data extracted from the time series with different in situ autonomic physiological signal data lengths could be used as the independent variables of a predictive model. As 32 samples of physiological data were not sufficient for the machine learning model, we increased the data by dividing it per session using only 32 participants’ intervention data and supplementing the insufficient data using SMOTE methodology. This technique can minimize performance degradation due to a lack of data. We generated cutoff values for the classification groups for labeling via k-means clustering. This is an unsupervised learning method based on the absence of a label to evaluate the model performance. We could explain which variable plays the most crucial role in predicting specific anxiety symptoms and VR sickness.

Among the anxiety symptoms related to the ISS, the most predictable was ISS mistake anxiety. Emotion comprised 5 main subsystems, namely, “cognitive component (appraisal), neurophysiological component (bodily symptoms), motivational component (action tendencies), motor expression component (facial and vocal expression), and subjective feeling component (emotional experience)” [46]. Shame is highly correlated with the body’s physiological responses because of its mechanism [47]. Mistake anxiety is the core symptom of SAD. The high predictability of these symptoms is related to the reliability of HR and GSR predictions of SAD symptoms.

Among the scales related to PERS, the most predictable was PERS positivity. Rumination may negatively affect physical and mental health [48,49]. However, this is inconsistent with previous studies reporting that positive rumination either is not associated with social anxiety or is low [28,30]. In contrast, other studies have reported that positive and negative rumination is high in social anxiety. Socially anxious individuals ruminate broadly, positively, or negatively about all aspects of social interactions when faced with ambiguity [50]. However, no previous study has examined the relationship between in situ autonomic physiological responses, and negative and positive rumination. Additional studies are required to determine whether these results are reproducible.

Several factors can cause VR sickness. Humans perceive their direction and movement through various sensory organs. Individuals may experience motion sickness if they repeatedly receive sensory information that differs from their prediction [51]. Visual movement may cause motion sickness [52,53]. When visual stimulation is the primary cause of motion sickness, it is called visually induced motion sickness. Previous studies have assessed virtual perceptions related to recognizing self-motion [54]. If the degree of physiological arousal (HR, blood pressure, skin conductance, respiration, skin temperature, and blood volume pulse amplitude) is high, the possibility of VR motion sickness is high [55].

We used HR and GSR for machine learning–based prediction in our study. These are important indicators of physiological arousal. SAD-related psychological symptoms and VR sickness during VR treatment can be determined in advance through the proposed model. The intervention of medical staff can also improve patient compliance. However, we must develop additional real-time measurement factors to better predict other VR sickness subdomain symptoms.

We predicted specific anxiety symptom severity and VR sickness severity via in situ physiological signal data from actual cases. We used supervised and unsupervised learning and data generation to build and evaluate the SAD and VR sickness predictive models. Considering these advantages, specific anxiety symptoms and VR sickness could be assessed more accurately. The data were labeled using an unsupervised learning method. After separating the severe and nonsevere groups, significant differences were found for each classification group using a *t* test. The real-time evaluation of VR motion sickness can help improve patient compliance with treatment. Moreover, we can reduce the time required for a survey by predicting participants’ anxiety using objective data. Predicting survey results using objective in situ autonomic physiological signal data makes less subjective intervention possible compared with the conventional survey-based method. In addition, the evaluation of symptoms through real-time autonomic physiological signals shows the possibility of evaluating psychiatric symptoms in real time to increase interactivity, an essential element in the personalized VR treatment process.

### Limitations

This study had several limitations. First, our study evaluated in situ autonomic physiological signals and symptom-related data in a SAD group alone. Subsequently, it included no control groups. This study used machine learning models to discriminate between severe and nonsevere groups because real-time physiological signal data were measured only for participants above a certain Korean Social Interaction Anxiety Scale (K-SAD) score. In some cases, after labeling the severe and nonsevere groups, there was no significant difference between groups. These results can be inferred as a limitation of the methodology that divides the group with a K-SAD score above 82 into severe and nonsevere groups according to specific anxiety symptoms. Although an unsupervised learning methodology distinguished respondents, it was challenging to explain clear criteria other than to the participants in the experiment. Second, this study used self-rating score-based scales, which could be associated with bias. Third, this study could only classify the severity of SAD (K-SAD ≥82) in the group with a particular score or higher. Fourth, there was insufficient evaluation of the response to a specific task or exposure situation during a VR session. Because insufficient data are obtained using a SMOTE model, there is a limitation in not using the actual data. In addition, it is unknown how performance changes as an individual progresses during a session. Furthermore, we cannot determine how in situ autonomic physiological signals change when a specific situation occurs in each session. Fifth, the number of participants who participated in the VR treatment session was 32, but the number of participants used for the analysis was 26 (a small sample size). The decrease in performance can be inferred to be limited to the improvement in performance because the number of individuals who participated in the VR sessions was small. Therefore, we need to carefully interpret the results and secure a larger number of samples for future studies. However, since it is challenging to obtain real-time in situ data, we believe that the strength of this study is that real valuable data from participants were utilized for analysis.

### Conclusion

This study showed that using in situ autonomous physiological signal data measured during a VR intervention can predict specific anxiety symptoms and VR sickness in patients with SAD. Using real-time physiological data from VR sessions, we can classify the severity of specific SAD symptoms and utilize the findings for personalized digital treatment. Machine learning models can assist in the decisions of medical staff and the construction of interactive VR treatment. Future research should focus on various predictive methodologies to enhance the tailored interactive function and to maximize the convenience of VR treatment. Additionally, to improve the clinical prediction performance and increase accuracy, more abundant and appropriate data will need to be collected.

## Appendix

Multimedia Appendix 1 Psychological scale prediction (linear regression model).

Multimedia Appendix 2 Correlation table for each feature.

Multimedia Appendix 3 Box plot of specific anxiety symptoms and virtual reality sickness severe and nonsevere group clustering results.

Multimedia Appendix 4 Results of specific anxiety symptoms.

Multimedia Appendix 5 Results of virtual reality sickness.

Multimedia Appendix 6 Receiver operating characteristic curve of specific anxiety symptom prediction models (logistic regression model, random forest model, and naïve Bayesian model).

Multimedia Appendix 7 Receiver operating characteristic curve of a virtual reality sickness prediction model (random forest model).

## Abbreviations

AUC: area under the curve
CBT: cognitive behavioral therapy
GSR: galvanic skin response
HR: heart rate
ISS: Internalized Shame Scale
K-SAD: Korean Social Interaction Anxiety Scale
PERS: Post-Event Rumination Scale
SAD: social anxiety disorder
SMOTE: Synthetic Minority Oversampling Technique
SSQ: Simulator Sickness Questionnaire
VR: virtual reality

## References

[1] Cannistraro P, Rauch S (2003). Neural circuitry of anxiety: evidence from structural and functional neuroimaging studies. Psychopharmacol Bull.

[2] Garfinkel SN, Eccles JA, Critchley HD (2015). The heart, the brain, and the regulation of emotion. JAMA Psychiatry.

[3] (2013). Diagnostic and Statistical Manual of Mental Disorders, Fifth Edition.

[4] Thurston RC, Rewak M, Kubzansky LD (2013). An anxious heart: anxiety and the onset of cardiovascular diseases. Prog Cardiovasc Dis.

[5] Friedman BH (2007). An autonomic flexibility-neurovisceral integration model of anxiety and cardiac vagal tone. Biol Psychol.

[6] Rapee RM, Heimberg RG (1997). A cognitive-behavioral model of anxiety in social phobia. Behaviour Research and Therapy.

[7] Heimberg RG (2002). Cognitive-behavioral therapy for social anxiety disorder: current status and future directions. Biological Psychiatry.

[8] Maples-Keller JL, Bunnell BE, Kim S, Rothbaum BO (2017). The use of virtual reality technology in the treatment of anxiety and other psychiatric disorders. Harv Rev Psychiatry.

[9] Geraets CN, Veling W, Witlox M, Staring AB, Matthijssen SJ, Cath D (2019). Virtual reality-based cognitive behavioural therapy for patients with generalized social anxiety disorder: a pilot study. Behav. Cogn. Psychother.

[10] McCauley ME, Sharkey TJ (1992). Cybersickness: perception of self-motion in virtual environments. Presence: Teleoperators & Virtual Environments.

[11] LaViola JJ (2000). A discussion of cybersickness in virtual environments. SIGCHI Bull.

[12] Kennedy RS, Lane NE, Berbaum KS, Lilienthal MG (1993). Simulator sickness questionnaire: an enhanced method for quantifying simulator sickness. The International Journal of Aviation Psychology.

[13] Sevinc V, Berkman MI (2020). Psychometric evaluation of Simulator Sickness Questionnaire and its variants as a measure of cybersickness in consumer virtual environments. Appl Ergon.

[14] Bouchard S, Dumoulin S, Robillard G, Guitard T, Klinger É, Forget H, Loranger C, Roucaut FX (2017). Virtual reality compared with exposure in the treatment of social anxiety disorder: a three-arm randomised controlled trial. Br J Psychiatry.

[15] Kim H, Lee S, Jung D, Hur J, Lee H, Lee S, Kim GJ, Cho C, Choi S, Lee S, Cho C (2020). Effectiveness of a participatory and interactive virtual reality intervention in patients with social anxiety disorder: longitudinal questionnaire study. J Med Internet Res.

[16] Lee H, Choi J, Jung D, Hur J, Cho C (2021). The effects of virtual reality treatment on prefrontal cortex activity in patients with social anxiety disorder: participatory and interactive virtual reality treatment study. J Med Internet Res.

[17] Yoo S, Kim Y, Noh J, Oh K, Kim C, NamKoong K, Chae J, Lee G (2006). Validity of Korean version of the mini-international neuropsychiatric interview. Anxiety and Mood.

[18] Hur J, Shin H, Jung D, Lee H, Lee S, Kim GJ, Cho C, Choi S, Lee S, Cho C (2021). Virtual reality-based psychotherapy in social anxiety disorder: fMRI study using a self-referential task. JMIR Ment Health.

[19] Powers MB, Briceno NF, Gresham R, Jouriles EN, Emmelkamp PMG, Smits JAJ (2013). Do conversations with virtual avatars increase feelings of social anxiety?. J Anxiety Disord.

[20] Anderson PL, Price M, Edwards SM, Obasaju MA, Schmertz SK, Zimand E, Calamaras MR (2013). Virtual reality exposure therapy for social anxiety disorder: a randomized controlled trial. J Consult Clin Psychol.

[21] Social Anxiety Disorder VR treatment. YouTube.

[22] Beck A, Epstein N, Brown G, Steer R (1993). Beck anxiety inventory. J of Consult Clin Psychol.

[23] Spielberger CD, Gonzalez-Reigosa F, Martinez-Urrutia A, Natalicio LF, Natalicio DS (1971). The state-trait anxiety inventory. Revista Interamericana De Psicología/Interamerican Journal of Psychology.

[24] Davidson J, Potts N, Richichi E, Ford S, Krishnan K, Smith R, Wilson W (1991). The Brief Social Phobia Scale. J Clin Psychiatry.

[25] Mattick RP, Clarke JC (1998). Development and validation of measures of social phobia scrutiny fear and social interaction anxiety. Behav Res Ther.

[26] Watson D, Friend R (1969). Measurement of social-evaluative anxiety. J Consult Clin Psychol.

[27] Cook DR (1988). Measuring shame. Alcoholism Treatment Quarterly.

[28] Abbott MJ, Rapee RM (2004). Post-event rumination and negative self-appraisal in social phobia before and after treatment. J Abnorm Psychol.

[29] Lee I, Choi H (2005). Assessment of shame and its relationship with maternal attachment, hypersensitive narcissism and loneliness. Korean Journal of Counseling and Psychotherapy.

[30] Edwards S, Rapee R, Franklin J (2003). Postevent rumination and recall bias for a social performance event in high and low socially anxious individuals. Cognitive Therapy and Research.

[31] Jain AK (2010). Data clustering: 50 years beyond K-means. Pattern Recognition Letters.

[32] Duarte M detecta: A Python module to detect events in data (Version v0.0.5). Zenodo.

[33] Chawla NV, Bowyer KW, Hall LO, Kegelmeyer WP (2002). SMOTE: Synthetic Minority Over-sampling Technique. jair.

[34] LaValley MP (2008). Logistic regression. Circulation.

[35] Pal M (2007). Random forest classifier for remote sensing classification. International Journal of Remote Sensing.

[36] Murphy KP (2006). Naive Bayes classifiers. University of British Columbia.

[37] Sokolova M, Japkowicz N, Szpakowicz S, Sattar A, Kang B (2006). Beyond Accuracy, F-Score and ROC: A family of discriminant measures for performance evaluation. AI 2006: Advances in Artificial Intelligence. AI 2006. Lecture Notes in Computer Science, vol 4304.

[38] Breiman L (2001). Random forests. Machine Learning.

[39] Gerlach AL, Mourlane D, Rist F (2004). Public and private heart rate feedback in social phobia: a manipulation of anxiety visibility. Cogn Behav Ther.

[40] Alvares GA, Quintana DS, Kemp AH, Van Zwieten A, Balleine BW, Hickie IB, Guastella AJ (2013). Reduced heart rate variability in social anxiety disorder: associations with gender and symptom severity. PLoS One.

[41] Rösler L, Göhring S, Strunz M, Gamer M (2021). Social anxiety is associated with heart rate but not gaze behavior in a real social interaction. J Behav Ther Exp Psychiatry.

[42] Wen W, Liu G, Mao Z, Huang W, Zhang X, Hu H, Yang J, Jia W (2020). Toward constructing a real-time social anxiety evaluation system: exploring effective heart rate features. IEEE Trans. Affective Comput.

[43] Zhang X, Wen W, Liu G, Hu H (2016). Recognition of public speaking anxiety on the recurrence quantification analysis of GSR signals.

[44] Park MJ, Kim DJ, Lee U, Na EJ, Jeon HJ (2019). A literature overview of virtual reality (VR) in treatment of psychiatric disorders: recent advances and limitations. Front Psychiatry.

[45] Verhoef REJ, van Dijk A, Verhulp EE, de Castro BO (2021). Interactive virtual reality assessment of aggressive social information processing in boys with behaviour problems: A pilot study. Clin Psychol Psychother.

[46] Scherer KR (2016). What are emotions? And how can they be measured?. Social Science Information.

[47] Hedman E, Ström P, Stünkel A, Mörtberg E (2013). Shame and guilt in social anxiety disorder: effects of cognitive behavior therapy and association with social anxiety and depressive symptoms. PLoS One.

[48] Brosschot JF, Verkuil B, Thayer JF (2010). Conscious and unconscious perseverative cognition: is a large part of prolonged physiological activity due to unconscious stress?. J Psychosom Res.

[49] Glynn LM, Christenfeld N, Gerin W (2002). The role of rumination in recovery from reactivity: cardiovascular consequences of emotional states. Psychosom Med.

[50] Dannahy L, Stopa L (2007). Post-event processing in social anxiety. Behav Res Ther.

[51] Sherman C (2002). Motion sickness: review of causes and preventive strategies. J Travel Med.

[52] Bonato F, Bubka A, Palmisano S, Phillip D, Moreno G (2008). Vection change exacerbates simulator sickness in virtual environments. Presence: Teleoperators and Virtual Environments.

[53] Keshavarz B, Riecke BE, Hettinger LJ, Campos JL (2015). Vection and visually induced motion sickness: how are they related?. Front Psychol.

[54] Lubeck AJ, Bos JE, Stins JF (2015). Motion in images is essential to cause motion sickness symptoms, but not to increase postural sway. Displays.

[55] Macedonio MF, Parsons TD, DiGiuseppe RA, Weiderhold BA, Rizzo AA (2007). Immersiveness and physiological arousal within panoramic video-based virtual reality. Cyberpsychol Behav.

