# Supplementary material for: Prediction of Specific Anxiety Symptoms and Virtual Reality Sickness Using In Situ Autonomic Physiological Signals During Virtual Reality Treatment in Patients With Social Anxiety Disorder: Mixed Methods Study
Source: JMIR Serious Games. 2022 Sep 16;10(3):e38284. doi: 10.2196/38284 (PMC9526108; doi:10.2196/38284)
Supplement: Multimedia Appendix 1 [file games_v10i3e38284_app1.pdf]

Multimedia Appendix 1

Psychological scale prediction (linear regression model)

Note: RMSE, Root Mean Square Error

feature	RMSE
Internalized Shame Scale mistaken anxiety	2.46
Internalized Shame Scale emptiness	3.70
Internalized Shame Scale self-punishment	4.24
Internalized Shame Scale inappropriate	6.46
Post-Event Rumination Scale positive	6.57
The Brief Fear of Negative Evaluation	8.08
Post-Event Rumination Scale	9.59
State-Trait Anxiety Inventory	9.65
Social Interaction Anxiety Scale	10.77
Liebowitz Social Anxiety Scale anxiety	11.06
Beck Anxiety Inventory	11.24
Liebowitz Social Anxiety Scale avoidance	11.48
Post-Event Rumination Scale negative	11.51
State-Trait Anxiety Inventory	11.65
Social Phobia Scale	12.05
Internalized Shame Scale	12.75
Korean version of Social Avoidance and Distress	16.51
