# Supplementary material for: Prediction of Specific Anxiety Symptoms and Virtual Reality Sickness Using In Situ Autonomic Physiological Signals During Virtual Reality Treatment in Patients With Social Anxiety Disorder: Mixed Methods Study
Source: JMIR Serious Games. 2022 Sep 16;10(3):e38284. doi: 10.2196/38284 (PMC9526108; doi:10.2196/38284)
Supplement: Multimedia Appendix 2 [file games_v10i3e38284_app2.pdf]

# Multimedia Appendix 2

## Correlation table for each feature

Note: HR, heart rate; GSR, Galvanic Skin Response; SD, standard deviation

| | | HR | | | | | | | | GSR | | | | | | |
|---|---|---|---|---|---|---|---|---|---|---|---|---|---|---|---|---|
| | | **average** | **SD** | **Min value** | **Max value** | **linear regression coefficient** | **total change** | **peak ratio** | **average change** | **average** | **SD** | **Min value** | **Max value** | **linear regression coefficient** | **total change** | **peak ratio** |
| **HR** | **SD** | 0.79 | | | | | | | | | | | | | | |
| | **min value** | 0.42 | 0.07 | | | | | | | | | | | | | |
| | **max value** | 0.55 | 0.67 | 0.23 | | | | | | | | | | | | |
| | **linear regression coefficient** | -0.26 | -0.41 | -0.16 | -0.23 | | | | | | | | | | | |
| | **total change** | 0.75 | 0.74 | 0.19 | 0.59 | -0.06 | | | | | | | | | | |
| | **peak ratio** | 0.73 | 0.48 | 0.62 | 0.52 | -0.15 | 0.61 | | | | | | | | | |
| | **average change** | 0.14 | 0.19 | -0.04 | -0.11 | -0.48 | -0.07 | 0.11 | | | | | | | | |
| **GSR** | **average** | 0.80 | 0.76 | 0.01 | 0.41 | -0.34 | 0.55 | 0.36 | 0.19 | | | | | | | |
| | **SD** | 0.72 | 0.83 | 0.06 | 0.50 | -0.40 | 0.61 | 0.40 | 0.09 | 0.85 | | | | | | |
| | **min value** | -0.20 | -0.17 | -0.05 | -0.18 | 0.09 | -0.15 | -0.17 | 0.01 | -0.04 | -0.20 | | | | | |
| | **max value** | 0.51 | 0.59 | 0.17 | 0.59 | -0.19 | 0.49 | 0.34 | -0.23 | 0.52 | 0.68 | -0.12 | | | | |
| | **linear regression coefficient** | -0.48 | -0.48 | -0.06 | -0.29 | 0.43 | -0.49 | -0.31 | -0.06 | -0.48 | -0.62 | 0.08 | -0.31 | | | |
| | **total change** | 0.73 | 0.57 | 0.08 | 0.36 | -0.24 | 0.51 | 0.33 | 0.09 | 0.78 | 0.63 | -0.17 | 0.51 | -0.32 | | |
| | **peak ratio** | 0.71 | 0.71 | -0.02 | 0.37 | -0.33 | 0.48 | 0.26 | 0.16 | 0.92 | 0.74 | 0.13 | 0.53 | -0.40 | 0.80 | |
| | **average change** | 0.08 | 0.19 | -0.06 | 0.03 | -0.52 | -0.10 | 0.01 | 0.74 | 0.22 | 0.12 | 0.09 | -0.14 | -0.13 | 0.10 | 0.21 |
