# Supplementary material for: Prediction of Specific Anxiety Symptoms and Virtual Reality Sickness Using In Situ Autonomic Physiological Signals During Virtual Reality Treatment in Patients With Social Anxiety Disorder: Mixed Methods Study
Source: JMIR Serious Games. 2022 Sep 16;10(3):e38284. doi: 10.2196/38284 (PMC9526108; doi:10.2196/38284)

Multimedia Appendix 3

Box plot of specific anxiety symptoms and virtual reality sickness severe and nonsevere group clustering results

Note: ISS, internalized shame scale; HR, heart rate; GSR, Galvanic Skin Response; SD, standard deviation

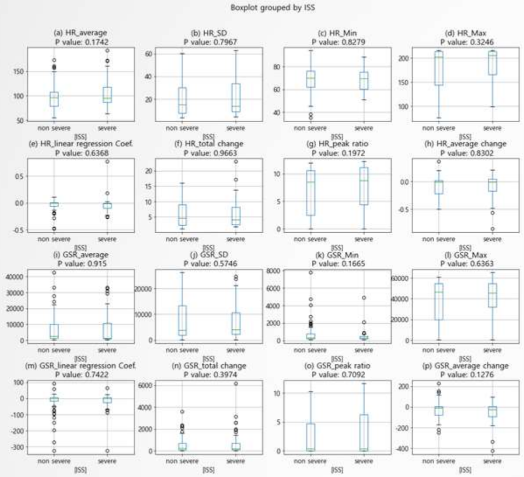

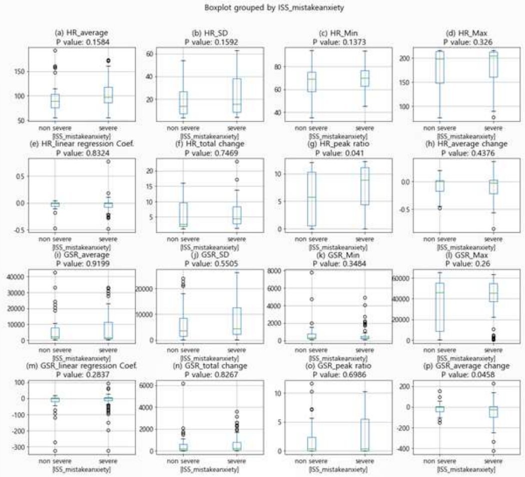

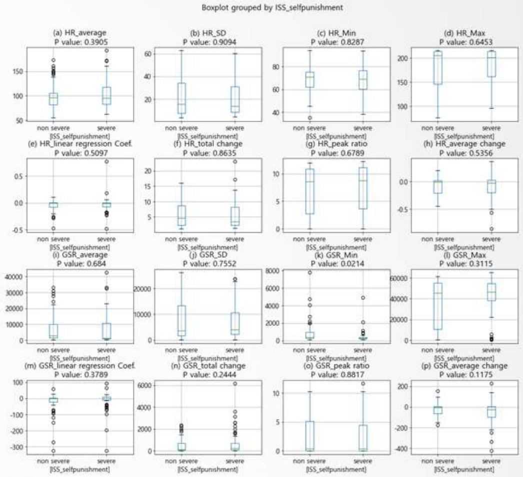

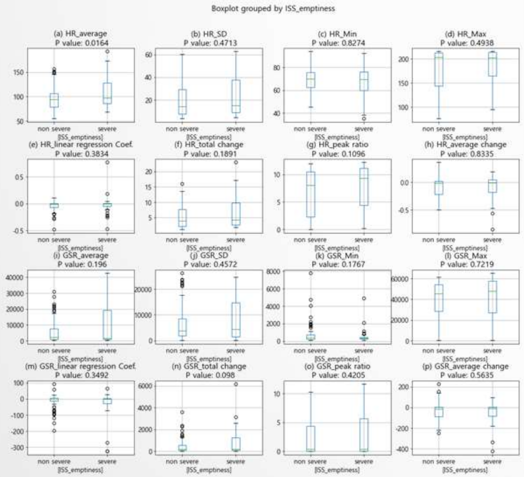

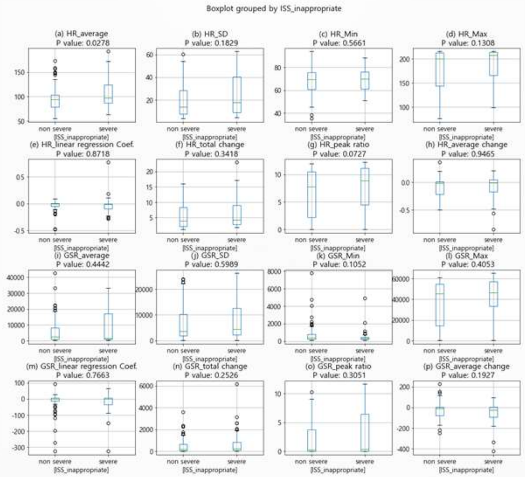

Multimedia Appendix 3

Box plot of specific anxiety symptoms and virtual reality sickness severe and nonsevere group clustering results

Note: PERS, Post-Event Rumination Scale; HR, heart rate; GSR, Galvanic Skin Response; SD, standard deviation

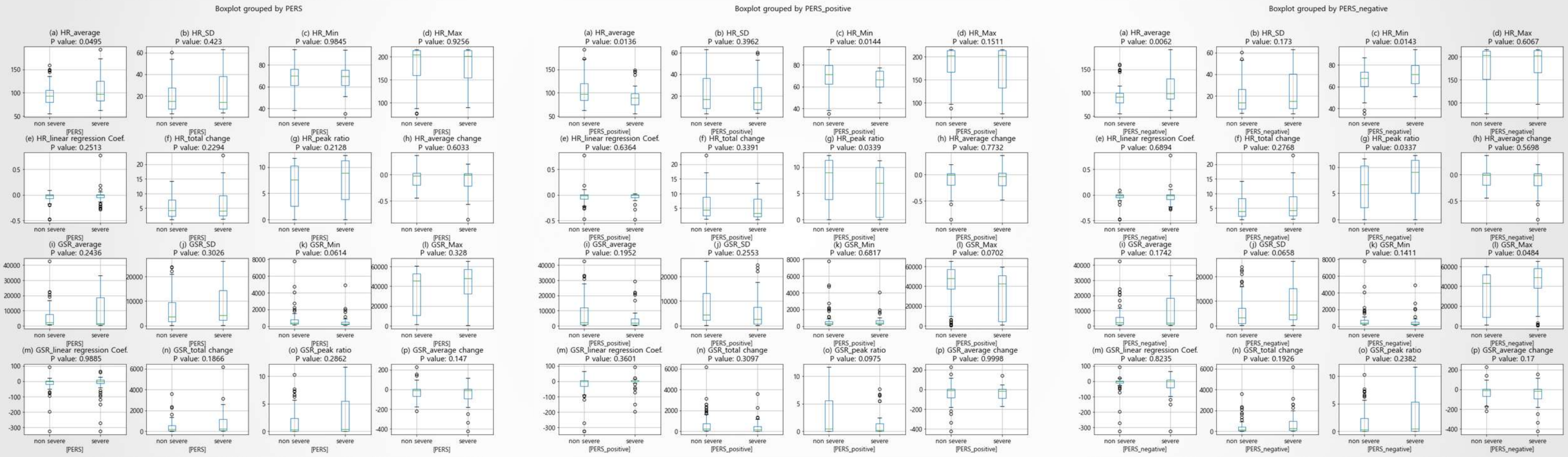

Multimedia Appendix 3

Box plot of specific anxiety symptoms and virtual reality sickness severe and nonsevere group clustering results

HR, heart rate; GSR, Galvanic Skin Response; SD, standard deviation

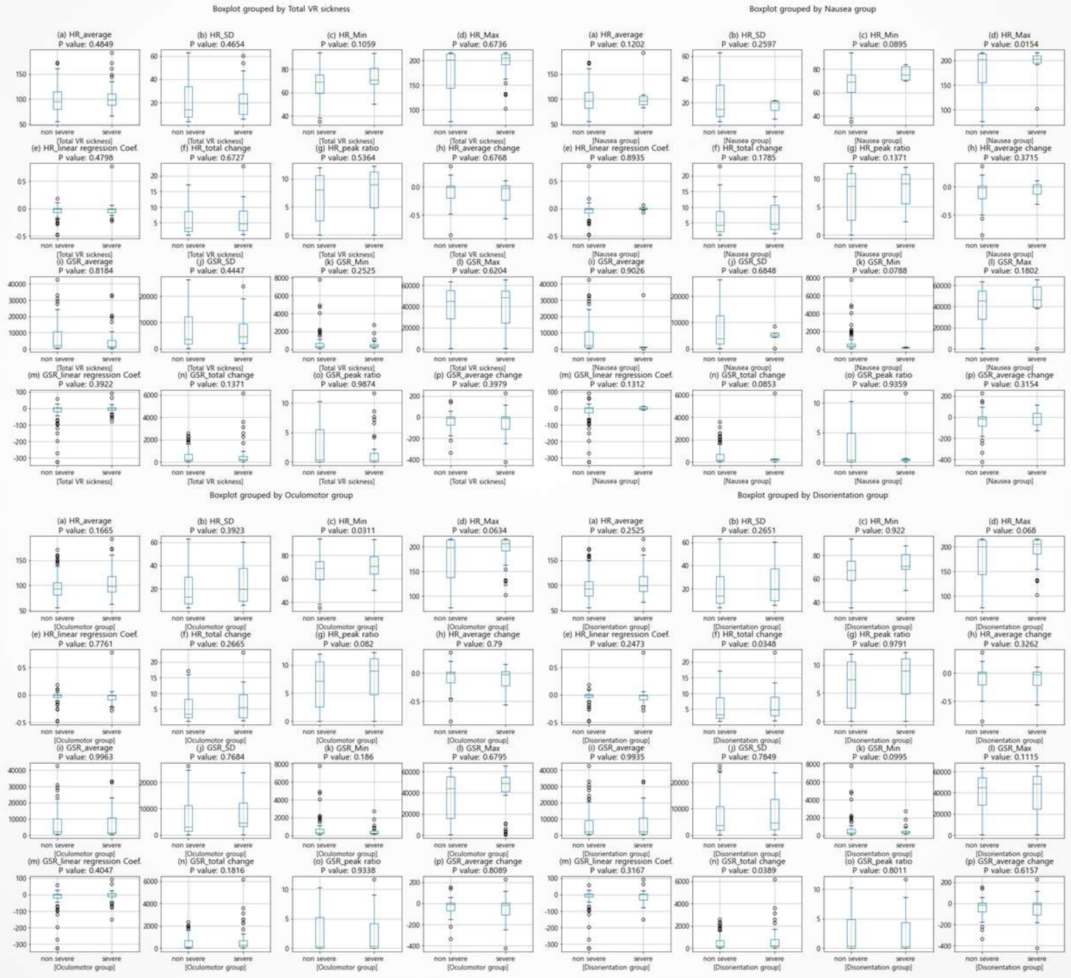

Supplement: Multimedia Appendix 3 [file games_v10i3e38284_app3.pdf]
