# Supplementary material for: Prediction of Specific Anxiety Symptoms and Virtual Reality Sickness Using In Situ Autonomic Physiological Signals During Virtual Reality Treatment in Patients With Social Anxiety Disorder: Mixed Methods Study
Source: JMIR Serious Games. 2022 Sep 16;10(3):e38284. doi: 10.2196/38284 (PMC9526108; doi:10.2196/38284)
Supplement: Multimedia Appendix 4 [file games_v10i3e38284_app4.pdf]

Multimedia Appendix 4

Results (t test) of specific anxiety symptoms

Note: ISS, Internalized Shame Scale; PERS, Post-Event Rumination Scale ; HR, heart rate; GSR, Galvanic Skin Response; SD, standard deviation

	ISS		ISS mistaken anxiety		ISS self-punishment		ISS emptiness		ISS inappropriate		PERS		PERS positive		PERS negative	
	t	p	t	p	t	p	t	p	t	p	t	p	t	p	t	p
**HR**																
**average**	-1.368	0.174	-1.421	0.158	-0.862	0.391	-2.441	0.016*	-2.232	0.028*	-1.987	0.050*	2.512	0.014*	-2.793	0.006**
**SD**	-0.258	0.797	-1.418	0.159	0.114	0.909	-0.723	0.471	-1.341	0.183	-0.804	0.423	0.852	0.396	-1.372	0.173
**min value**	-0.218	0.828	-1.498	0.137	-0.217	0.829	0.219	0.827	-0.576	0.566	0.019	0.985	2.489	0.014*	-2.491	0.014*
**max value**	-0.99	0.325	-0.987	0.326	-0.462	0.645	-0.687	0.494	-1.523	0.131	0.094	0.926	1.446	0.151	-0.516	0.607
**linear regression coefficient**	-0.474	0.637	-0.212	0.832	-0.662	0.51	-0.875	0.383	-0.162	0.872	-1.154	0.251	0.474	0.636	-0.401	0.689
**total change**	0.042	0.966	-0.324	0.747	0.172	0.864	-1.322	0.189	-0.955	0.342	-1.209	0.229	0.961	0.339	-1.093	0.277
**peak ratio**	-1.298	0.197	-2.07	0.041*	-0.415	0.679	-1.614	0.11	-1.813	0.073	-1.254	0.213	2.15	0.034*	-2.153	0.034*
**average change**	0.215	0.83	0.779	0.438	0.622	0.536	0.211	0.833	0.067	0.947	0.521	0.603	-0.289	0.773	0.57	0.57
**GSR**																
**average**	0.107	0.915	-0.101	0.92	0.408	0.684	-1.302	0.196	-0.768	0.444	-1.173	0.244	1.304	0.195	-1.368	0.174
**SD**	0.563	0.575	-0.599	0.55	0.313	0.755	-0.746	0.457	-0.528	0.599	-1.036	0.303	1.144	0.255	-1.86	0.066
**min value**	1.393	0.167	0.942	0.348	2.337	0.021*	1.36	0.177	1.635	0.105	1.892	0.061	0.411	0.682	1.483	0.141
**max value**	-0.474	0.636	-1.133	0.26	-1.017	0.312	-0.357	0.722	-0.836	0.405	-0.983	0.328	1.83	0.07	-1.998	0.048*
**linear regression coefficient**	-0.33	0.742	-1.078	0.284	-0.884	0.379	0.94	0.349	-0.298	0.766	0.014	0.989	-0.919	0.36	0.224	0.824
**total change**	-0.85	0.397	-0.219	0.827	-1.171	0.244	-1.67	0.098	-1.151	0.253	-1.33	0.187	1.021	0.31	-1.312	0.193
**peak ratio**	-0.374	0.709	-0.388	0.699	0.149	0.882	-0.809	0.421	-1.031	0.305	-1.072	0.286	1.672	0.098	-1.186	0.238
**average change**	1.536	0.128	2.022	0.046*	1.579	0.117	0.58	0.563	1.311	0.193	1.461	0.147	0.000	1.000	1.382	0.17
