# Supplementary material for: Prediction of Specific Anxiety Symptoms and Virtual Reality Sickness Using In Situ Autonomic Physiological Signals During Virtual Reality Treatment in Patients With Social Anxiety Disorder: Mixed Methods Study
Source: JMIR Serious Games. 2022 Sep 16;10(3):e38284. doi: 10.2196/38284 (PMC9526108; doi:10.2196/38284)
Supplement: Multimedia Appendix 5 [file games_v10i3e38284_app5.pdf]

Multimedia Appendix 5

Results (t test) of virtual reality sickness

Note: HR, heart rate; GSR, Galvanic Skin Response; SD, standard deviation

	Total VR sickness		Nausea group		Oculomotor group		Disorientation group	
	t	p	t	p	T	p	t	P
**Heart rate**								
**average**	-0.701	0.485	-1.567	0.120	-1.394	0.166	-1.151	0.253
**SD**	0.733	0.465	-1.133	0.260	-0.859	0.392	-1.121	0.265
**min**	-1.632	0.106	-1.714	0.090	-2.185	0.031*	0.098	0.922
**max**	-0.422	0.674	-2.465	0.015*	-1.877	0.063	-1.844	0.068
**Coef.**	-0.709	0.480	-0.134	0.894	-0.285	0.776	-1.163	0.247
**abs change**	-0.424	0.673	-1.355	0.178	-1.117	0.267	-2.139	0.035*
**peak**	-0.620	0.536	-1.499	0.137	-1.757	0.082	0.026	0.979
**avg change**	-0.418	0.677	0.898	0.372	0.267	0.790	0.987	0.326
**GSR**								
**average**	0.230	0.818	-0.123	0.903	-0.005	0.996	0.008	0.993
**SD**	0.767	0.445	-0.407	0.685	-0.295	0.768	0.274	0.785
**min**	1.151	0.253	1.775	0.079	1.332	0.186	1.662	0.100
**max**	-0.497	0.620	-1.349	0.180	-0.414	0.679	-1.605	0.112
**Coef.**	-0.859	0.392	-1.521	0.131	-0.837	0.405	-1.006	0.317
**abs change**	-1.498	0.137	-1.738	0.085	-1.345	0.182	-2.092	0.039*
**peak**	0.016	0.987	0.081	0.936	0.083	0.934	-0.253	0.801
**avg change**	-0.849	0.398	1.009	0.315	0.243	0.809	0.503	0.616
