# Supplementary material for: Prediction of Specific Anxiety Symptoms and Virtual Reality Sickness Using In Situ Autonomic Physiological Signals During Virtual Reality Treatment in Patients With Social Anxiety Disorder: Mixed Methods Study
Source: JMIR Serious Games. 2022 Sep 16;10(3):e38284. doi: 10.2196/38284 (PMC9526108; doi:10.2196/38284)

Multimedia Appendix 6

Receiver operating characteristic curve of specific anxiety symptom prediction models (logistic regression model)

Note: Note: ISS, Internalized Shame Scale; PERS, Post-Event Rumination Scale; ROC, Receiver Operating Characteristic;

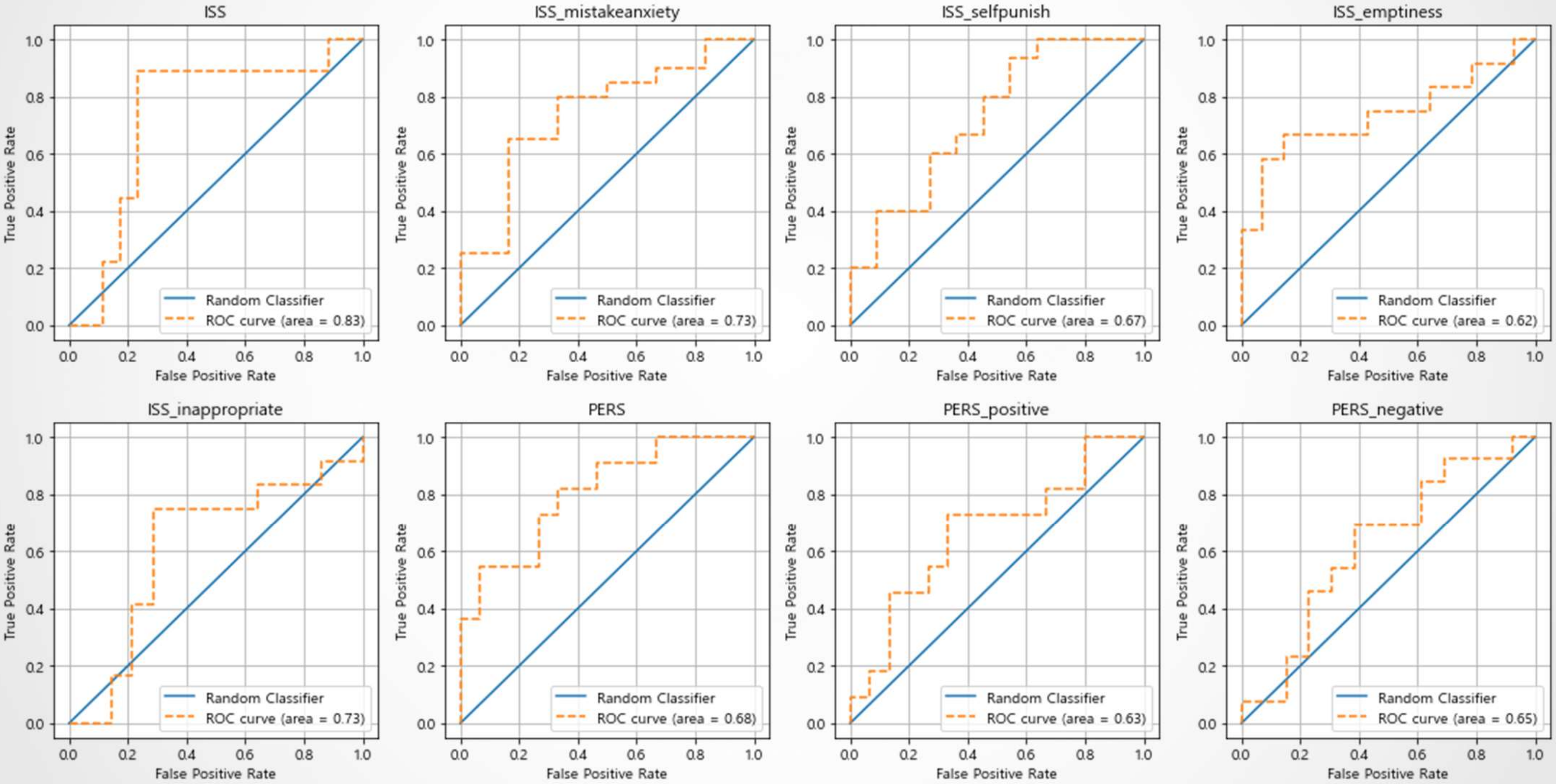

Multimedia Appendix 6

Receiver operating characteristic curve of specific anxiety symptom prediction models (random Forest model)

Note: Note: ISS, Internalized Shame Scale; PERS, Post-Event Rumination Scale; ROC, Receiver Operating Characteristic;

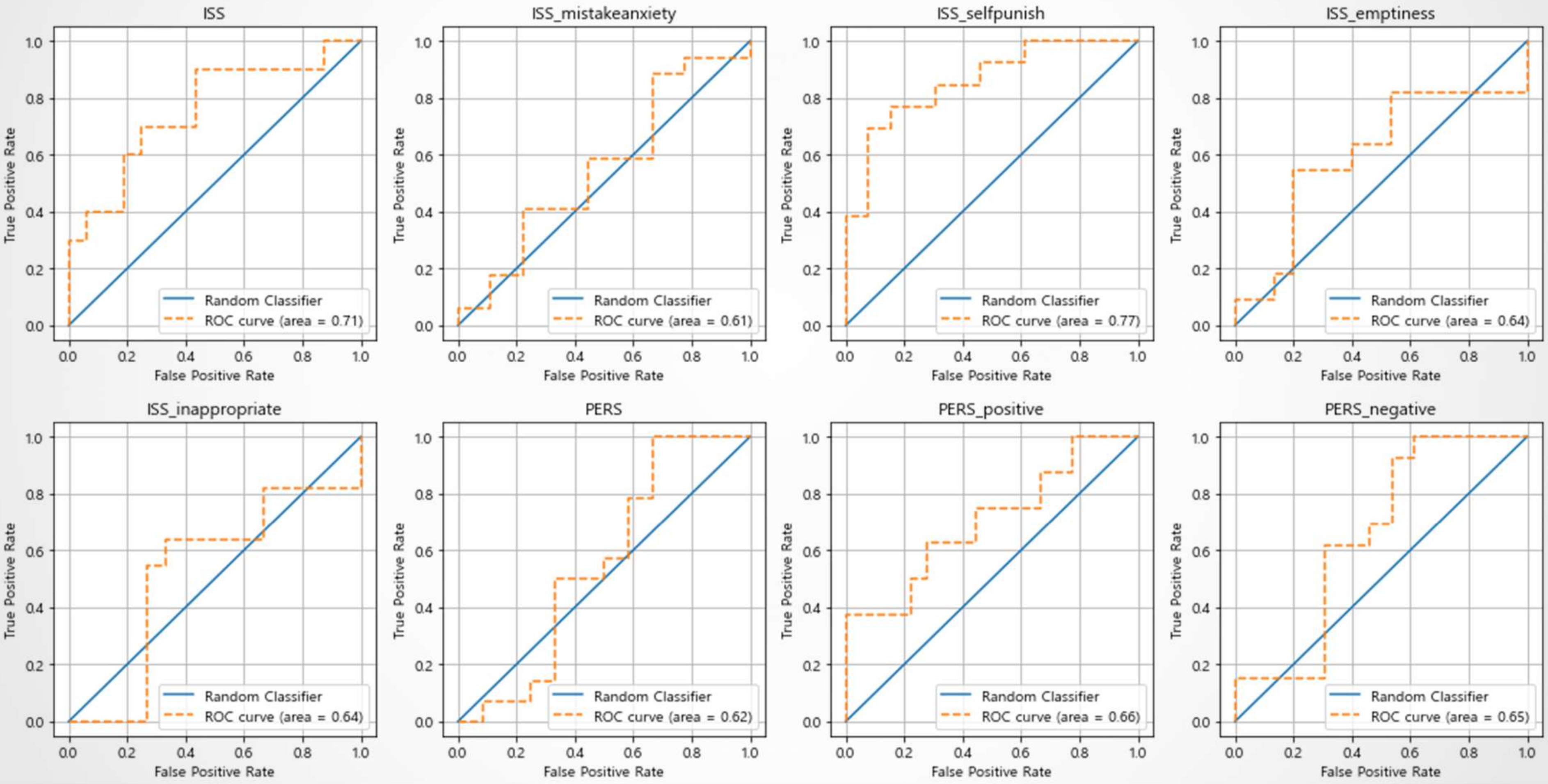

Multimedia Appendix 6

Receiver operating characteristic curve of specific anxiety symptom prediction models (naïve Bayesian model)

Note: Note: ISS, Internalized Shame Scale; PERS, Post-Event Rumination Scale; ROC, Receiver Operating Characteristic

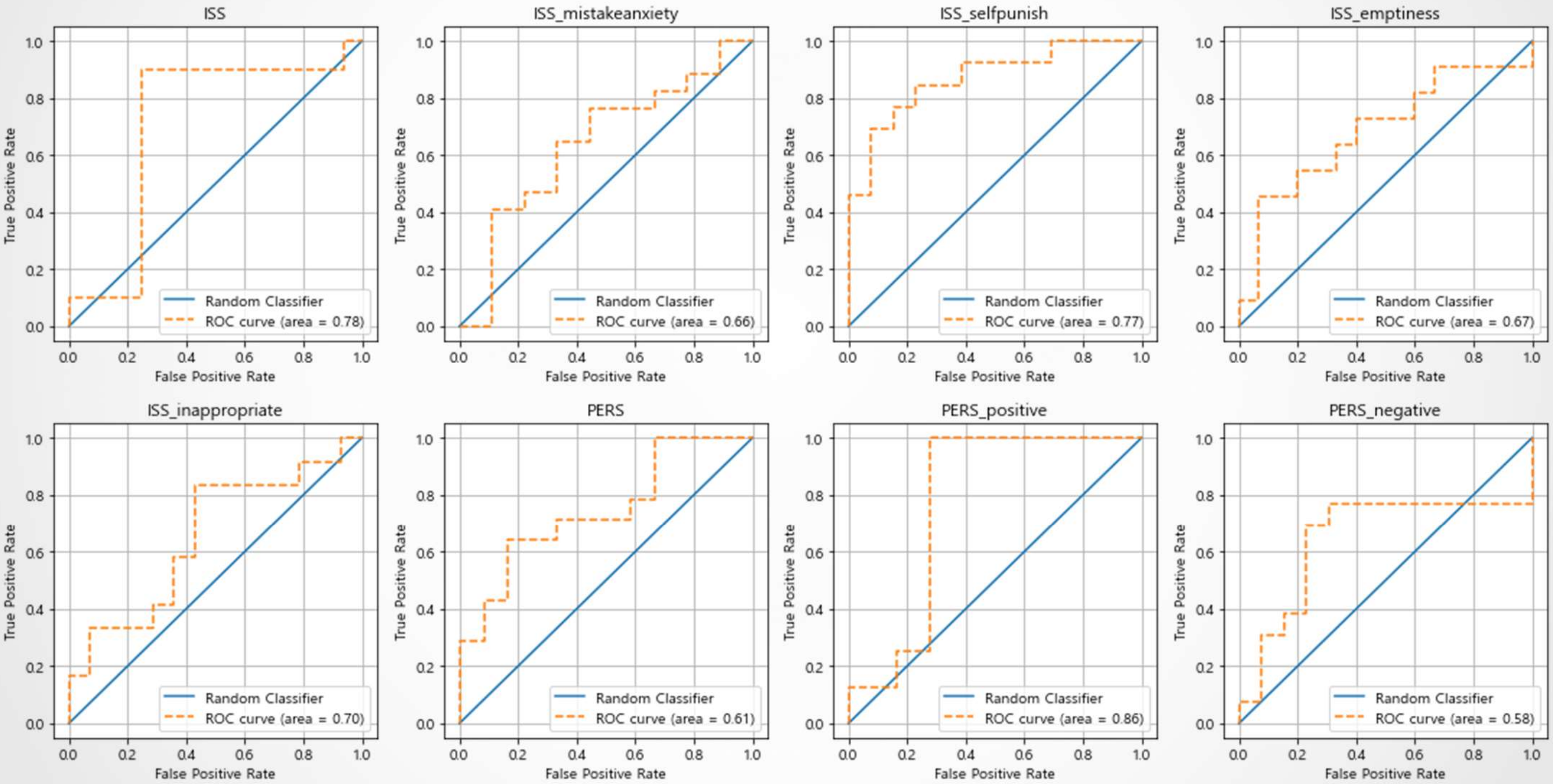

Supplement: Multimedia Appendix 6 [file games_v10i3e38284_app6.pdf]
