# Supplementary material for: Prediction of Specific Anxiety Symptoms and Virtual Reality Sickness Using In Situ Autonomic Physiological Signals During Virtual Reality Treatment in Patients With Social Anxiety Disorder: Mixed Methods Study
Source: JMIR Serious Games. 2022 Sep 16;10(3):e38284. doi: 10.2196/38284 (PMC9526108; doi:10.2196/38284)

Multimedia Appendix 7

Receiver operating characteristic curve of a virtual reality sickness prediction model (random forest model)

Note: Note: VR, Virtual Reality; ROC, Receiver Operating Characteristic

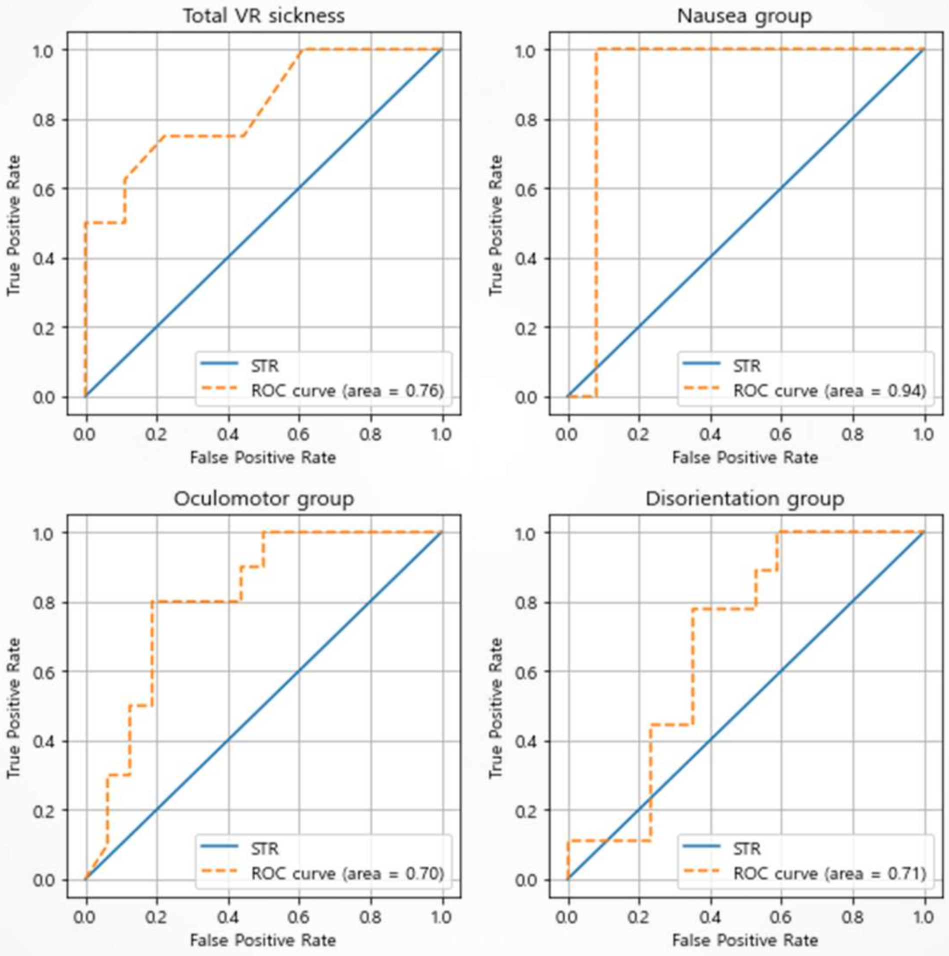

Supplement: Multimedia Appendix 7 [file games_v10i3e38284_app7.pdf]
